# Correspondence

**Published:** 1901-05

**Authors:** S. H. Swain

**Affiliations:** Decatur, Ill.


### CORRESPONDENCE.

DECATUR, ILL., April 1, 1901.

EDITOR JOURNAL COMPARATIVE MEDICINE, ETC.:

ON page 949 of the March issue of the *American Veterinary Review* Dr. James Robertson, of and for the State Board of Veterinary Medical and Surgical Examiners, attempts to defend himself and the board against a series of wholesale charges, specifications, and positive proof which I preferred against them in the January number of your JOURNAL. And now, while I feel like apologizing to the profession, one and all, for asking them to suffer the infliction of carefully reading that weak deliverence from his pen in the March issue of the *Review*, I do so only because it is supposed to be the best defence that can come from that more or less respected, august, and erudite body; but why they presume to put forth even this bastard and brainless defence is a mystery to me. The board admits it has never denied a certificate to any applicant, however undeserving and degraded, if only he had the fee. It admits having licensed an individual from the insane asylum; it pleads guilty to having passed an applicant whom our committee found unable to answer a single primary question, of which fact I refer to the Illinois Veterinary Medical and Surgical Association in proof. The board admits telling these applicants, against whom plentiful and persistent protests were made, that they need not feel uneasy about losing the $20 fee in case they failed to pass, because the board would pass them; and the board always thus exchanged the license for the fee. I say the board admits the truth of all these allegations, because they are not denied. The board does not pretend to deny or palliate this infamous practice, because it cannot. From all over the State would concentrate clouds of witnesses to confound them. They must take their medicine. When conscience and common honesty compelled our committee to condemn this board and its infamous actions, they answer back that we are "now graduates," and, when we adduce evidence that many of the world's most eminent men belong to our contemptible class, they call us "egotist," seeking the association of these giants of earth who walk the mountain ranges of the world When we prove they have licensed men that are demented they say that "dementia is much in evidence," whatever that silly phrase may mean, and insinuate a suspicion of our sanity. When we affirm the fact that we and others protested the unfitness to practice of these applicants, the board did not deny that fact, but sought to deceive the profession by publishing, as a letter of protest, what was in reality an expression of my utter disgust with the board for its cringing, cowardly, and contemptible conduct, in dereliction of its plain and positive duty. Dr. Robertson knew when he published that letter as a letter of protest from me that he was deliberately trying to deceive the profession at my expense. This act of shameless duplicity, while not open, perhaps, to the charge of downright lying, is such a cunning, careless, contemptible twisting and torturing of the truth that in courtesy to him I will call his conduct cowardly prevarication.

He has there on file two thoroughbred letters of vigorous protest from me, dated July 28 and December 16, 1899, which the board will never publish. I think I have answered Dr. Robertson's deliverances fully and fairly. He says he "shot at random at our committee and hit the King Bee of the hornets' nest and hurt him badly." In behalf of our committee I may say his assault was like bombarding a stone wall with mush poultices. If our committee's report was false it was calumny and should have called out their cannon; but when we smote them on one side they turned the other, and when we apply the lash they offer no resistance, but simply lie down and seek to be funny. His defence of our arrangement, either as a literary or logical fulmination, is a complete, flat, and foolish failure. He purposely perverts certain parts and passages of my article, seeking in the absence of originality to steal his neighbor's thunder in search of lightning. Doctor, when in battle never go over to the enemy to borrow ammunition.

But, to be candid, I must confess the doctor's article, however soft and senseless, is vastly better than I expected, for you know "Ex nihilo nihil fit"—"Out of nothing, nothing can come." The doctor once expressed a haughty, imperious, and blood-curdling contempt for the whole kit and clan of us poor plebeian non-graduates, but he says now he has the profoundest respect for most of them—except me. He pities me for allowing the lurid scintillations of my cerebrum to shatter the vase and letting the overflowing fountain of my "undoubted talent run wild." Now, is it not true that a recent rasping the doctor received from us had some potency in pumping into him some of this "profound respect" for my recently despised but now profoundly respected compeers? I have stated in this article that this State Examining Board admits having licensed men to practice who are absolutely unqualified. With their qualifications this board claims to have nothing to do. In the name of outraged decency and honor, and in behalf of the profession at large, I say, Shame on such official infamy! Mr.

Editor, I will prove every allegation I have uttered and truthfully paint this incompetent board so black that the aroused profession must remove them.

In answer to my letter of December 16, 1899, in which I severely criticised the board for its shameful conduct, I have the secretary's answer defending the board's action. He does not deny granting licenses to every applicant, but says the law, prior to January 1, 1900, compels them so to do, provided the applicant proves that he has practised three years or has a diploma. Thus armed and barring only by immoral character, this guileless goodly board says it is compelled to grant licenses to every applicant or be subjected to mandamus process, compelling it to violate the trust imposed in it. Such is the board's interpretation of the law, by which he justifies the board's guilt prior to 1900. "But," he says, "after the time limit (January 1, 1900) of this law then rest assured that not only non-graduates, but all holders of diplomas from two-year colleges will be compelled to come before the board and pass the examination as required by the law." Holy Moses! I said. Here is a virtuous deliverance that could only come from an immaculate conception. Prior to January 1, 1900, the board claimed that the law bound them to license every applicant, and they did so freely—for the fee—but the law explicitly states that an examination before this board of examiners touching the applicant's qualifications to practice veterinary medicine and surgery in the State of Illinois "shall be discretionary with the board." This the secretary of the board denies; hence their wholesale licensing, and cowardly cringing before every illiterate tramp that came before them holding up the fee.

We respectfully submit that the law not only permitted but made it the imperative duty of this board to examine these men under the law prior to the time limit when they had a suspicion of the applicant's utter incompetency, and especially when such suspicion was reduced to a certainity by protests being filed against them by many of the best members of the profession in the State. Such was the board's status under the old law or prior to January 1, 1900. Now, what would any competent and conscientious examining board do in the presence of these applicants, these facts, and these protests? What would I do? I would say, "Gentlemen, I am here to safeguard the highest interests of the veterinary medical and surgical profession in the State of Illinois. Prior to January 1, 1900, the law leaves it optional with the board whether it shall license you, after three years' practice, without an examination; but against each of you, gentlemen, there are lodged and on file here some very emphatic protests from the profession against your being licensed, and thus the board by this act of the profession must exercise its discretion and subject each of you to an examination.

But the board after January 1, 1900, as we have noted, promised, in effect, "never to be caught in another scheme or scrape and to never let another guilty man escape." The Bible declares, "He that knoweth his duty and doeth it not shall be beaten with many stripes." But they grew gradually and greatly worse, fell from grace, lost their religion, and became so ungodly that they actually sold licenses to practice veterinary medicine and surgery to every trifling devil that applied and paid the fee. This latter was the "Alpha and Omega," the beginning and the end, the first and the last, and the *sine qua non*—that is, without which there is nothing. Judas Iscariot sold the Saviour for thirty pieces of silver, but this board sold out the veterinary profession in the State of Illinois for twenty pieces. Judas had the decency to go out and hang himself, but this board hesitates even after our committee has made its duty plain. When the devil took the Saviour up on the mountain and offered him all the kingdoms and glory of the world if he would set aside principles and fall down and worship him, what do you think, under the circumstances, would have been the status of this board? Why, every mother's son would have gone down on his knees for the first time in his life, with hands up, offering to sell his soul to the devil for half the sum. My reason for expressing this belief is because I know they have sold themselves to a bigger fool than the devil for a devilish sight smaller sum.

Notwithstanding all this, there is something about this board we must admire. Under the terrible rain of shot and shell our committee was forced to focus upon them they only squirmed and grinned, and when we uncovered and began to dissect them they good-naturedly made fun of their own funeral. Against this board personally I cannot feel the slightest prejudice or displeasure. I doubt not they are the mildest-mannered men that ever "scuttled ship or cut a throat;" and as husbands, fathers, and friends they may be faithful, they may be even elegant, ideal, and an ornament to any social circle; but while all this may be true, the same could be said of King Charles I., and yet the good people of England were reluctantly compelled to depose him from office and cut off his head because his official conduct was a curse to the State. We recommend this board to the clemency of the outraged profession of the State.

Respectfully,

S. H. Swain, V.S.

